# Effect of salt stress on proximate composition of duckweed (*Lemna minor* L.)

**DOI:** 10.1016/j.heliyon.2021.e07399

**Published:** 2021-06-24

**Authors:** Hafiz Ullah, Bakhtiar Gul, Haroon Khan, Umar Zeb

**Affiliations:** a Department of Botany, University of Chitral, KP, Pakistan; b Department of Weed Science, Faculty of Crop Protection Sciences, The University of Agriculture Peshawar, Pakistan; c Key Laboratory of Resource Biology and Biotechnology, College of Life Sciences, Northwest University, China

**Keywords:** Proximate analysis, Salinity, *Lemna minor* L., Nutritional value, Aquatic weeds

## Abstract

The shortage of conventional feedstuff is one of the rising issues faced by the developing countries of the world. To bridge the gap between supply and demand of the major feedstuff it is desirable to practice the use of non-conventional feed resources. Duckweeds are the aquatic macrophytes growing in stagnant water bodies that offer a choice to be used as an alternate feed. Before the use of any alternate feed, it is vital to know the nutritional composition of the feed under diverse environmental conditions. The objective of this study was to investigate the influence of salinity, abiotic stress, on the proximate composition of duckweed (*Lemna minor* L.). The experiment was laid out in Completely Randomized Design (CRD) with 3 repeats. Data was collected on protein, lipid, carbohydrate, and mineral contents. In the laboratory trial plants were grown under the saline condition of different concentrations ranging from 2 g NaCl L^−1^ to 12 g NaCl L^−1^ for a growing period of 20 days. The biomasses obtained were tested for proximate composition. ANOVA of the result exhibited a significant effect of salinity on the proximate composition of the plant. Protein residues of the plant started declining above the concentration of 4 g NaCl L^−1^ until the lowest value was obtained at 12 g NaCl L^−1^. Lipid composition showed more sensitivity to the stress with a sharp decline above 2 g NaCl L^−1^ having a minimum value at 12 g NaCl L^−1^. Carbohydrate contents increased with increasing salinity up to 6 g NaCl L^−1^ above which a decrease was observed. The highest accumulation of the macronutrients i.e., Ca, Mg, took place in the lower range of concentration of the salt. The percentage compositions of micronutrients such as Fe, Mn, and Zn percentage were reduced at a higher range of salinity while the optimum level was recorded in plants treated with 2 g NaCl L^−1^, followed by control. The total accumulation of both macro and micronutrients was higher in the plant material treated with a lower level of salt concentration, concluding a significant effect of salinity on proximate composition. As for the Indus water salinity level, the plant has the capacity of tolerance and can be grown without affecting its proximate composition.

## 1 Introduction

In a developing country like Pakistan, the foremost limitation in raising livestock and poultry is the fragile availability of nutrients. Rangelands and pastures which could serve as a reservoir for nutrients are subjected to deformation due to overuse and lack of strategies for sustainable productivity. There is a limited knowledge of the use of non-conventional feed resources to improve livestock nutrition. The unit area serving the purpose of fodder production has shrunk in the recent past because of growing infrastructure. Livestock raising policies in Pakistan do not support vertical expansion of the livestock sector. Pakistan annually requires about 10.9 and 90.36 million tons of crude protein (CP) and total digestible nutrients (TDN) to feed animals. However, the availability of these two nutrients is 6.7 and 69.0 million tons. The gulf between demand and supply is widening day by day and thus, CP and TDN are 38.10 and 24.02% underprovided per year. Presently, the major sources of nutrients supply are green fodders, crop residues, grazing vacant lands, post-harvest grazing, vegetable farms, and meals, respectively. The issue of the growing demand for nutrient supply could be resolved through effective fodder policies and the use of non-conventional feedstuff (Sarwar et al., 2002).

Crop production statistics show that in Pakistan indigenous feed resources are short for livestock and poultry requirements. The supply and demand gap for dry biomass, crude protein (CP), and metabolizable energy (ME) are 19.4%, 37.2%, and 38.0%, respectively. Residues of crops are the most widely used feed comprising 58.8% of the total feed input, while fodder and grazing contribute 23.8% and 9.2%, correspondingly. Grains and by-products contributed 8.2% to the feed supply. To bridge the gap between supply and demand, enormous quantities of oilseed meals are imported particularly for the poultry sector. Keeping in view the fast expansion in poultry, dairy, and feedlot farming in Pakistan, the feed demand will further enlarge, and this calls future attention to efficient and intensive utilization of the local non-conventional feed resources (Habib et al., 2016).

The minimal availability and rising cost of feed constituents, especially high-quality animal protein has captured the attention of animal nutritionists towards alternative source of protein in diet formulation. There are some unconventional resources of proteins, carbohydrates, and minerals that can effectively be used as animal feed to get products with good nutritive value. Duckweed (*Lemna minor* L.) is one of them, which offers a cheaper source of feed in many developing countries. *L. minor* is among the smallest angiosperms. It reproduces mostly vegetatively through budding in a short span of about two days if the environmental conditions are favorable (Journey et al., 1993).

Duckweeds are small (3.5–6.5 mm and does not exceed 10 mm) free-floating hydrophytes with worldwide distribution. They are classified as aquatic macrophytes belonging to the family Lemnaceae. Duckweed (*L. minor)* is potential nutrient absorbers, mitigates nutrient enrichment and eutrophication, and releases oxygen during photosynthesis. This plant can effectively be used for scavenging toxic metals and other nutrients in the wastewater and thus can be applied for the phytoremediation of industrial effluents before discharging the water into any freshwater body. Duckweeds show enormous growth capability in freshwater and make a thick green blanket on stagnant or slow-moving water. It can grow both in fresh and brackish water (Zimmo 2003).

The most common environmental factors which influence the growth of duckweed are the twin problems of water and salt stress. Some macrophytes tolerate salinity while others are sensitive to the stress and cannot survive above 50–100 mM NaCl (Downton and Läuchli 1984). Salinity is an abiotic stress and exerts both osmotic and toxic effects on plants. In aquatic plants, salinity decreases water potential and hence limits the absorption of adequate level of water needed for the physiological processes of the plant (Taiz and Zeiger 1991). The following effect is specific toxicity which hinders uptake of nutrients, causes membrane damage, and adversely affects cellular metabolism (Volkmar et al., 1998). Growth of *L. minor* decreases markedly with increasing concentrations of NaCl and mannitol. The lower concentrations of NaCl (50 mM) and mannitol (100 mM) started to reduce growth significantly in the later stages of growth, while the higher concentrations (100 and 200 mM) reduce growth from the beginning (Wendeou et al., 2013).

Crop production in Pakistan is mostly confined to the Indus Basin which faces the problem of salinity and waterlogging. The rivers and their tributaries containing saltwater salinize the irrigation system of the country. Currently, 131Bn m^3^ of water runs in the irrigation system diverted from the river Indus. The Indus water increments the soil with 33 metric tons of salt. This indicates an annual addition of 16.6 metric tons of salts to the Indus basin. The basin faces the twin problems of salinity and waterlogging where optimal crop production can hardly be practiced. The process of secondary salinization and shallow groundwater aggravate the conditions even more for ordinary agricultural practices. Salt-loaded soil and water bodies have become the major ecological constraints to agriculture. An estimated 6 million hectare is already under stress mostly restricted to regions under irrigation (Qureshi et al., 2004). About 1 million hectares of the land are water-logged (Haider et al., 1999). Such waterlogged saline soil can be brought under non-conventional cultivation through aquatic macrophytes like the duckweed (*L. minor).*

*L. minor* is effective in absorbing organic and inorganic nutrients from wastewater of ponds, ditches and sewerage water. The use of aquatic plants as feed ingredient at low levels in poultry diet has shown remarkable outcomes, especially in the production of egg pigments and broiler skin (Haustein et al., 1990). High protein-containing *L. minor* resemble soyabean meal (Porath et al., 1979). Among the factors which often affect the nutritional composition of duckweeds are plant species, season of the year, temperature salinity, mineral contents of water particularly N and P are more significant. Duckweed is rich in essential amino acids that have a balanced level of lysine (6.9 g/1000 g protein), an amino acid deficient in other plant proteins (Porath et al., 1979). It is also a cheap source of minerals and pigments like beta-carotene and xanthophylls (Journey et al., 1993). In comparison to other terrestrial plants, it contains ten times higher beta-carotene and xanthophylls concentration (Mbagwu and Adeniji 1988).

Duckweeds serve as a potential feed ingredient in poultry. Higher weight gain in chicks up to 3 weeks of age took place when fed with dehydrated duckweed up to 5 percent level of mixed feed (Truax et al., 1972). Sewage grown duckweeds have been successfully used as a substitute for Soyabean meal fish meal up to 15% in the diet of poultry for good egg production and high yolk production. The use of duckweed as poultry feed has been established by many authors (Islam et al., 1997; Leng et al., 1995; Samnang, 1999). Crude protein contents of duckweed and amino acid balance along with vitamins and minerals make the plant an ideal candidate for animal feed (Landolt and Kandeler 1987; Men et al., 2001).

Being a reliable source of nutrients, *L. minor* can be utilized for poultry as well as fish feed with a dual benefit of weed management, biomass disposal, and protein supplement in the feed where it is easily available. The present study was conducted to pursue the following objectives:

1. To evaluate the effect of salinity levels on the nutritional contents of *L. minor*, and to discern the favorable and inhibitory range of salinity for the plant growth.
2. To investigate the feasibility of the culture of the plant in brackish water bodies in Pakistan.
3. To recommend the critical salinity range to produce *L. minor* under the climatic conditions of Pakistan.

## 2 Materials and methods

### 2.1 Experimental site description

An experiment was conducted to evaluate the effect of various levels of salinity on the protein, lipid, carbohydrate, and mineral contents of duckweed (*L. minor*). The experiment was carried out in the Department of Weed Science, the University of Agriculture Peshawar, during March 2017, using Completely Randomized Design (CRD) with treatments replicated three times. Each pot was 21 inches in diameter with a depth of 10 inches. A fresh biomass of the plant was collected from local freshwater bodies. A measured biomass of 10 g of the plant was transferred to each pot already amended and labeled with treatments i.e. (2, 4, 5, 6, 8, 10, 12 g salt concentration) and control, in 3 replications. Data was recorded on the following parameters adopting the method given below for each parameter:

#### 2.1.1 Protein contents

Protein contents were investigated by using the Kjeldahl method. A known mass of oven-dried sample was oxidized in sulphuric acid (H_2_SO_4_) by using a catalyst like (CuSO_4_). All nitrogen present in the sample was converted into ammonium sulfate (NH_4_)_2_SO_4,_ the inorganic form. On reaction with NaOH, ammonium sulfate was released and distilled as ammonium hydroxide which was then accumulated in boric acid (BO_3_) solution followed by titration with standard solution as shown by the following equation (Hach et al., 1985).

Powdered sample + H_2_SO_4_ → (NH_4_)_2_ SO_4_ + CO_2_ + H_2_O

(NH_4_)_2_SO_4_ + 2NH_4_OH + 2NaO → NH_4_OH + Na_2_SO_4_

2NH_4_OH + H_3_BO_3_ → (NH_4_)_2_BO_3_ + 2H_2_O

(NH_4_)_2_BO_3_ + HCl → 2NH_4_Cl + H_3_BO_3_

**Calculations**

The percent nitrogen and crude protein was calculated as follows:

$$\text{\% N }=\frac{\left(\text{ S}-\text{B }\right)\times 0.014\times \text{ D }\times 100}{\text{Wt}.\text{ of sample }}$$

S = Volume of standard acid used for sample titration
B = Volume of standard acid used for blank titration
D = Sample dilution after digestion (volume ml)
∗0.014 is the milliequivalent weight of nitrogen

#### 2.1.2 Lipid content

Lipid was extracted by subjecting the samples to the Soxtec apparatus comprising an Extraction Unit and a Control Unit. The Extraction Unit was installed in a well-ventilated fume hood (airflow 0.5 ms^−1^). A (0.5 g) sample to be analyzed was weighed into thimbles and inserted in the Extraction Unit, containing extraction flasks. One-third of the flask was filled with petroleum ether which was used as a solvent. The flasks were heated by the electrical heating plate. The three-step extraction procedure consists of boiling, rinsing, and recovery. First, the solvent was heated. The heated solvent dissolved crude fat from the sample. The solvent was then heated to evaporate and recover by condensation. The flasks were dried at 100 °C for one hour. The flasks were weighed again to calculate the difference in weight (Thiex et al., 2003).

**Calculations**

Weight (g) of sample used for extraction = W_1_
Weight (g) of flask without fat = W_2_
Weight (g) of flask with fat = W_3_
Extractable fat (%) = $\frac{{\text{W}}_{3}-{\text{W}}_{2}}{{\text{W}}_{1}}\times \;100$

#### 2.1.3 Carbohydrate contents

Carbohydrate contents were investigated by using the following formula:

% Carbohydrate content = 100 g Dry sample − (Mineral content + Protein + Lipids) × 100

#### 2.1.4 Mineral contents

Mineral contents were investigated by the AA-7000 (Shimadzu) Atomic Absorption in the Department of Water Management Science, the University of Agriculture, Pakistan. The wet ashing/Acid digestion method was used for sample preparation. The digestion was made in Perchloric Acid. Digestion was followed by filtration with a glass filter and 100 ml volume was made with distilled water (Ghaedi et al., 2007).

Mineral (X) concentration = ((AAS result) X volume of sample) X dilution factor/weight of sample

### 2.2 Determination of salinity of Indus water

The Indus river basin is the major system of irrigation in Pakistan. In Pakistan, the basin extends from the Himalayas in the north to the plains of Sindh in the south. The river finally makes its way to the Arabian sea. Indus basin, which spread over an area of 520,000 km^2^ and constituted sixty-five percent of the surface area of Pakistan. The basin is the main source of water in Pakistan and all the irrigation channels flow from it. The salinity level of Indus basin tributaries was calculated through EC measurement using Adwa (AD 330) EC/TDS meter. The following conversion formula was used to calculate g/L of NaCl;

Total dissolved NaCl = EC × 640 (EC 0.1 to 5 ds/m)

### 2.3 Analysis of the data

The data were analyzed by using computer packages Mstat-C GenStat software by using the LSD test at 0.05 level of probability (Steel and Torrie 1980).

## 3 Result and discussion

### 3.1 Protein content (%)

Salinity showed a marked effect on protein contents of the *L. minor* as indicated by (Table 1). ANOVA of the result shows a significant variation in protein percentage with variation in concentrations of salt. Minimum protein content (25%) was obtained for treatments like 10 gL^-1^ and 12 gL^-1^ each. The highest protein of (33%) and (34%) proteins were recorded for a lower range of concentrations like 2 gL^-1^ and 4 gL^-1^ respectively. Protein contents in control remained statistically like lower concentrations like 2 gL^-1^ and 4 gL^-1^ respectively. Higher protein contents, in plants grown in the lowest concentrations of salt, prove that salinity offers stress to protein synthesis. Protein synthesis is one of the complex metabolic processes embracing different factors. If the stress is prolonged it could cause a decline of protein synthesis (Caplan et al., 1990). Ayala-Astorga and Alcaraz-Meléndez (2010) also demonstrated reduced protein content for *Paulownia imperialis* grown in high sodium chloride concentration. One of the metabolic constraints established by higher salt concentration is nitrogen metabolism, a significant factor in protein synthesis. The activity of nitrate reductase declines under saline conditions (AbdElBaki et al., 2000; Flores et al., 2000). A correlation exists between Cl^−1^ concentration in the external medium and the activity of nitrate reductase (Cram 1973; Smith 1973; Deane-Drummond and Glauss 1982; Flores et al., 2000). Elevation of salt concentration causes the removal of potassium from plant roots, necessary for protein synthesis. Soussi et al. (1999) stated that salinity retards nitrogen metabolism by shrinking the activity of nitrogenase in chickpea (*Cicer arietinum* L.). Reduction in nitrogen fixation due to salinity has also been reported in many legumes (Bekki et al., 1987). Nodulated roots of soybean, common bean, and alfalfa when subjected to salt stress show reduced growth of nodules and the plant. The plants also showed reduced nitrogenase activity (Serraz et al., 1998).

### 3.2 Lipid content (%)

Lipids are the most efficient source of stored food energy. They not only at as a source of energy but also play a functional role, thus protecting the sensitive internal organs of the plant and acting as key building blocks of cellular membranes (Singh et al., 2002). Data obtained from the experiment were analyzed to evaluate the effect of salinity levels on the lipid content of *L. minor* as presented in (Table 1). Maximum lipid content (7.33%) was exhibited by plants grown in control while minimum (2.66%) lipid content was extracted from plants grown in 12 g L^−1^ solution. A progressive decrease in lipid content percentage was evaluated with an increase in salt level. This indicates the inhibitory effect of high salt concentration on lipid metabolism. Lipids are important constituents of cell which play a pivotal role in permeability of cell (Bybordi et al., 2010). Under the condition of stress, a decrease happens in lipid metabolism. Increasing soil salt concentration strongly influences the biosynthesis of the essential lipid (Solinas et al., 1996). Hassanein (1999) testified that increasing salt concentration reduces the percentage of lipids in peanut (*A. hypogaea* L.) NaCl stress causes a change in lipid composition in marsh grass with a significant decrease in the percentage of sterols and phospholipids (Kerkeb et al., 2001).

### 3.3 Carbohydrate contents (%)

Statistical analysis of the data shows that the different salinity levels have a significant effect on the carbohydrate content of duckweed. The percentage of carbohydrate content percentage after application of different salt concentrations is shown in (Table 1). The highest percentage of (60.3%) and (60%) was obtained for 2 gL^-1^ and 4 gL^-1^ salt concentrations, respectively. A decrease in carbohydrate content was seen with an increase in salt concentration until the minimum value of 50% was attained for the highest salt concentration (12 gL^-1^). Growth reduction and low carbohydrate content is the result of physiological process. A decrease in carbohydrate contents with increasing salinity may be a direct or an indirect effect of the stress and cannot be attributed to a single factor. Long duration exposure of plants to salt stress decreases carbon assimilation because leaves get loaded with salt (Munns and Termatt 1986). A change in the percentage of carbohydrates with variation in salinity is partly because of low water potential in the cells which, in turn, causes stomatal closure and limits CO_2_ assimilation. The characteristic problem offered by salt stress is that it causes physiological drought and limits adequate water availability for carbohydrate metabolism. Many of the studies suggest that the process of photosynthesis is inhibited by salinity (Chaudhuri and Choudhuri 1997; Soussi et al., 1998; AliDinar et al., 1999; Romeroaranda et al., 2001; Kao et al., 2001). There are also reports that the rate of photosynthesis is enhanced by low salt concentration. Our study is in harmony with the findings of (Rajesh et al., 1998; Kurban et al., 1999). In legumes, it has been observed that CO_2_ assimilation is stimulated at a lower concentration of salts. In the same way, intercellular CO_2_ concentration decreases when plants are exposed to salinity (Kurban et al., 1999). During salt stress plant carries out photosynthesis with a low quantity of chlorophyll content resulting in minimum carbon fixation. The rate of respiration increases during stress conditions which ends up with a low rate of photosynthetic assimilates (Khavarinejad and Chaparzadeh 1998).

**Table 1:** Protein, lipid, and carbohydrate contents of duckweed (*L. minor*) after treatment with different salt concentrations.

Treatments (NaCl)	Proximate Composition (g/100 g)		
	Protein	Lipid	Carbohydrate
2 gL^-1^	33.0 ± 0.8^a^	5.33 ± 0.94^b^	60.33 ± 1.7^a^
4 gL^-1^	34.0 ± 0.8^a^	5.00 ± 0.81^b^	61.00 ± 1.5^a^
6 gL^-1^	27.0 ± 0.8^b^	4.67 ± 0.94^b^	58.33 ± 0.1^ab^
8 gL^-1^	29.6 ± 2.0^bc^	4.33 ± 0.95^bc^	56.33 ± 0.1^bc^
10 gL^-1^	25.0 ± 1.6^c^	4.33 ± 0.95^bc^	53.33 ± 0.1^cd^
12 gL^-1^	25.0 ± 0.8^c^	2.67 ± 0.47^c^	51.00 ± 0.4^d^
Control	32.66 ± 2.0^b^	7.33 ± 0.46^a^	59.33 ± 0.4^ab^
LSD at (p ≤ 0.05)	3.009	1.709	3.840

Means in the above with different subscripts significantly different (p ≤ 0.05) using LSD test.

### 3.4 Mineral contents (mg/100 g)

A higher rate of absorption of (NaCl) causes an imbalance in the mineral absorption of the plant. Duckweed plants after treatment with different salt concentrations were tested to evaluate the percentage composition of five minerals like Ca, Mg, Fe, Mn, and Zn. The result obtained s discussed as follows.

#### 3.4.1 Calcium (Ca^2+^)

With the increase in the level of salinity, the percentage composition of (Ca^2+^) declines as indicated in Table 2. Analysis of the data shows the maximum calcium (18 mg/100 g) in plants treated with the lowest salt concentration (2 gL^-1^). There exists an inverse relationship between salt concentration and (Ca^2+^) content of the plant. In many plants elevated treatment of salt increases the level of (Na^+^) and (Cl^−^) and reduces the (Ca^2+^) content (Khan et al. 1999, 2000; Khan 2001) In some plants like marine macroalgae, increased content of (Na^+^) and (Cl^−^) stimulate proline level but decrease the activity of proline dehydrogenase and (Ca^2+^) content showing loss of (Ca^2+^) because of accumulation of proline (Lee and Liu 1999). In mangrove species like (*B. parviflora* Roxb.), a decrease in (Ca^2+^) has been reported indicating membrane stability and a decrease in chlorophyll content (Parida et al., 2004). The reduced content of (Ca^2+^) might be the result of the suppressive effect of Na^+^ and K^+^ ions or it may be the result of ineffective transport of (Ca^2+^).

#### 3.4.2 Magnesium (Mg^+2^)

Table 2 shows the percentage of (Mg^+2^) in milligrams per 100 g of the dry biomass. The higher (34.3 mg/100 g) Mg^+2^ was noticed in plants grown in control followed by the second-highest percentage of (27.7 mg/100 g) for plants grown in treatment 2 gL^-1^ of salt concentration. Here like Ca^+2^ the inverse relationship exists between the two variables i.e., (Mg^+2^) content and salt concentration. In Chickpea (*Cicer arietinum* L.) increase in salinity checks the percentage composition of (Mg^+2^). A 21.5% and 37.1% decrease in (Mg^+2^) content was recorded in different cultivars of the plant. The study suggests that such a decline might be due to the suppressive effect of Na^+^ and K^+^ on the cation or it may be the result of inefficient transportation of the cation (Varsheny et al., 1998). Bhatt et al. (2008) suggested a decrease in (Mg^+2^) concentration in leaves of the genus *Ziziphus* with an increase in salt concentration. A decrease in (Mg^+2^) concentration might be the result of solute imbalance as well as reduced root growth under salinity. Talei et al. (2012) studied the reduction of mineral content upon application of salinity in (*Andrographis paniculata* Nees.) and concluded that the level of (Mg^+2^) decreases with increasing concentration of salts. The importance of magnesium cannot be negated in plant growth and development. Besides the role of (Mg) in chlorophyll structure and as an enzyme cofactor another essential role of (Mg) in plants is the export of photosynthates, which when impaired culminates into degradation of chlorophyll in (Mg) deficient leaves, resulting in higher oxygenase activity of RuBP carboxylase (Marschner and Cakmak 1989).

#### 3.4.3 Iron (Fe^+2^)

Data analysis of the effect of salt level on mineral contents is statistically significant. Upon chemical analysis of the percentage composition, a maximum (Fe^+2^) content (48.6 mg/100 g) was obtained from plants grown in control, without any salt treatment (Table 2). Next to this value is (41.7 mg/100 g) of the dry biomass obtained for treatment 2 gL^-1^. Our result shows a reduction in (Fe^+2^) content with an increase in the salinity level. The plants grown under salinity accumulate low concentrations of micronutrients in their biomasses (Page et al., 1990). Our results are at par with El-Bassiouny (2005), Sadak et al. (2010), Abdelhamid et al. (2010), and Taie et al. (2013). In stressed faba been it was observed that treatment with Nicotinamide led to the increase in minerals in the because of their role in osmotolerance including their absorption from soil. This decrease might be the outcome of the antagonism shown by Na^+^ to micronutrients. The experiment also showed a contrary relationship between (Fe) content and salinity. A minimum (Fe^+^) content of (31.7 g/100 mg) was obtained for higher concentration like 10 gL^-1^, indicating low (Fe^+^) content at higher salinity. Turan et al. (2010) also showed that high salinity causes a decrease of (Fe^+^) contents in the shoot of maize.

**Table 2:** Macro and micronutrient contents of duckweed (*L. minor*) after treatment with variable salt concentrations.

Treatment (NaCl)	Proximate Composition of Nutrients (mg/100 g)				
	Ca^+2^	Mg^+2^	Fe^+2^	Mn^+2^	Zn^+2^
2 gL^-1^	18 ± 0.03^a^	27.7 ± 0.38^b^	41.7 ± 0.08^b^	3.09 ± 0.04^a^	0.08 ± 0.04^b^
4 gL^-1^	6 ± 0.30^d^	24.7 ± 0.16^bc^	41.7 ± 0.08^b^	1.4 ± 0.08^c^	0.07 ± 0.04^c^
6 gL^-1^	6 ± 0.30^d^	18.3 ± 0.16^d^	37 ± 0.04^c^	0.6 ± 0.08^e^	0.07 ± 0.04^c^
8 gL^-1^	6 ± 0.30^d^	21.7 ± 0.16^cd^	37 ± 0.04^c^	0.5 ± 0.08^ef^	0.06 ± 0.04^d^
10 gL^-1^	9 ± 0.35^c^	19.3 ± 0.09^d^	31.7 ± 0.04^d^	0.3 ± 0.08^f^	0.06 ± 0.04^d^
12 gL^-1^	12 ± 0.23^c^	22.7 ± 0.04^bcd^	34.7 ± 0.03^cd^	0.89 ± 0.08^d^	0.05 ± 0.04^e^
Control	15 ± 0.16^b^	34.3 ± 0.03^a^	48.6 ± 0.04^a^	1.79 ± 0.08^b^	0.09 ± 0.04^a^
LSD (p ≤ 0.05)	0.002	0.005	0.003	0.0004	0.00005

Means with ±SD in the above with different subscripts significantly different (p ≤ 0.05) using LSD test.

#### 3.4.4 Manganese (Mn^+2^)

A significant effect of salt stress has been observed on the percentage composition of the mineral after analysis of the experimental data. The highest percentage of (0.0031% Mn^+^) is coming for plants treated with 2 (gL^−1^) concentration of NaCl, succeeded by control (no salt treatment). According to some workers, there exists a complex relationship between salinity and micronutrient concentration of different plants. In plants like barley, sugar beet, tomato salinity increases the concentration of Mn but reduces the concentration of the same mineral in squash, pea, and corn (Hu and Schmidhalter 2001). A decrease in Mn content is reported in barley (*Hordeum vulgare* L.), grown under saline condition. An addition of Mn in the solution under salt stress enhances the tolerance level of barley (Cramer and Nowak 1992). Most workers have mentioned that salinity results in the decline of Mn shoots. Examples are provided in bean (Doering et al., 1984), corn (Rahman et al., 1993) and squash, *Cucurbita pepo* L. (Maas et al., 1972). Some investigations show that Salinity either had no effect or increased Mn content in leaf or tissue. Our result agrees with the previous studies where salinity lowered Mn content in the plant material.

#### 3.4.5 Zinc (Zn^+2^)

The maximum value of zinc percentage was calculated to be (0.00009%) for control succeeded by the second-highest percentage of (0.00008%) for 2 gL^-1^ concentration. Therefore, it appears that salinity and zinc accumulation have a negative relationship. Our result is in alliance with the findings of (Turan et al., 2010), which also show a low accumulation of zinc during salt stress in maize. The availability of the nutrients depends on the twin factors of pH and pE of the medium as well as the nature of binding sites of the organic molecules to which the ions bind. The solubility of the micronutrients is predominantly low in saline and sodic soils and plants growing in such conditions have a deficiency of micronutrients (Fe, Mn, and Zn) in their biomass (Page et al., 1990). Variation in the accumulation of trace elements may exist depending upon the type of plant, type of tissue, growth period, and salinity level (Grattan and Grieve 1999). Some of the studies suggest no effect of salinity on the accumulation of Zn (Izzo et al., 1991) while other studies suggest a decrease of Zn content in the biomass of plants, like cucumber (Al-Harbi 1995). Our result shows a deficiency and is in close agreement with the latter.

### 3.5 Salt concentration of Indus basin

Indus basin makes the major irrigation system of Pakistan. Fed by the Indus river, it makes 65% of the country's surface land. The Indus river system serves the purpose of interconnection between the glaciers in the mountain and the water in the plains of Punjab and Sindh provinces of the country (Ojeh, 2006). The tributaries of the Indus basin were tested for their salt concentration. River Kabul, River Swat, River Chenab, River Jhelum, and River Chenab were the rivers from which samples were collected to measure EC. The level of salinity and TD increases down the terrain till it reaches the maximum of 1.7 g L^−1^ near Karachi. The purpose of this research is to test the level of salinity that can affect the organic composition of the duckweed and whether the plant can be grown along the Indus basin. The calculated data suggests that the duckweed, *Lemna minor,* can tolerate the Indus water salinity and can be grown without a significant change in its organic composition.

## 4 Conclusion

Duckweed (*Lemna minor*) is a plant that can be grown in ditches, ponds, swamps, and standing water bodies in Pakistan. The proximate composition of the plant shows that it can be used as an alternate source of feed for poultry and livestock, to meet the growing demand of the agriculture sector. Different salinity levels tested show that salt stress has a significant effect on various components of the proximate analysis like protein, lipid, carbohydrate, and mineral contents of the plant. The plant can fairly adjust the salinity level up to 6 (gL^−1^) without much change in proximate composition. Therefore, this plant can be grown in the standing water bodies of the Indus basin which has a salinity level of 1.7 g L^−1^. To sum up, the plant is appropriate to be grown in standing saline water bodies in Pakistan and can be adopted as an alternate source of feed for livestock and poultry.

## Declarations

### Author contribution statement

All authors listed have significantly contributed to the investigation, development and writing of this article.

### Funding statement

This research did not receive any specific grant from funding agencies in the public, commercial, or not-for-profit sectors.

### Data availability statement

Data will be made available on request.

### Declaration of interests statement

The authors declare no conflict of interest.

### Additional information

No additional information is available for this paper.
